# Fine Structure in Isotopic Peak Distributions Measured Using Fourier Transform Ion Cyclotron Resonance Mass Spectrometry: A Comparison between an Infinity ICR Cell and a Dynamically Harmonized ICR Cell

**DOI:** 10.1021/jasms.2c00093

**Published:** 2022-06-28

**Authors:** Jingsha Xu, Meng Li, Bryan Marzullo, Christopher A. Wootton, Mark P. Barrow, Peter B. O’Connor

**Affiliations:** Department of Chemistry, University of Warwick, Coventry CV4 7AL, United Kingdom

## Abstract

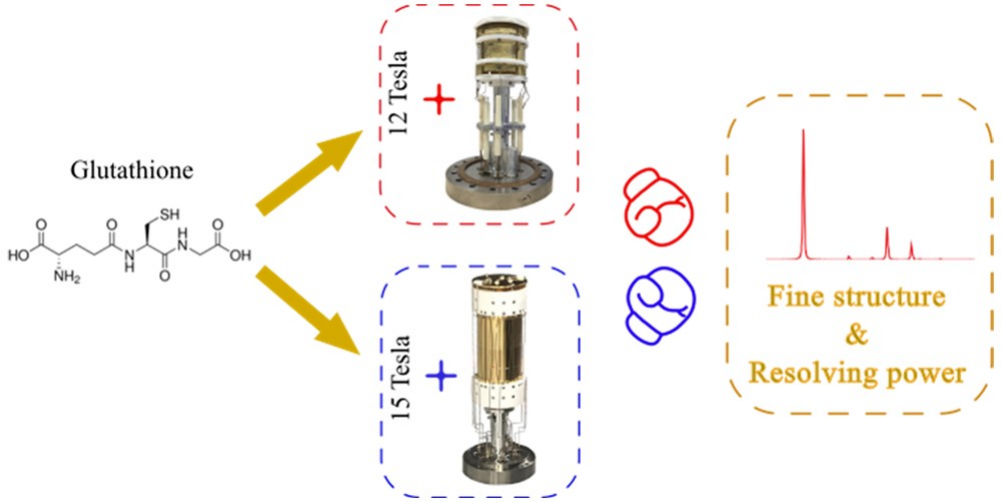

The fine structure of isotopic peak distributions of glutathione in mass spectra is measured using Fourier transform ion cyclotron resonance mass spectrometry (FT-ICR MS) at 12 and 15 T magnetic field, with an infinity cell and a dynamically harmonized cell (DHC) respectively. The resolved peaks in the fine structure of glutathione consist of ^2^H, ^13^C, ^15^N, ^17^O, ^18^O, ^33^S, ^34^S, ^36^S, and combinations of them. The positions of the measured fine structure peaks agree with the simulated isotopic distributions with the mass error less than 250 ppb in broadband mode for the infinity cell and no more than 125 ppb with the DHC after internal calibration. The 15 T FT-ICR MS with DHC cell also resolved around 30 isotopic peaks in broadband with a resolving power (RP) of 2 M. In narrowband (*m*/*z* 307–313), our current highest RP of 13.9 M in magnitude mode was observed with a 36 s transient length by the 15 T FT-ICR MS with the DHC and 2ω detection on the 15 T offers slightly higher RP (14.8 M) in only 18 s. For the 12 T FT-ICR MS with the infinity cell, the highest RP achieved was 15.6 M in magnitude mode with a transient length of 45 s. Peak decay was observed for low abundance peaks, which could be due to the suppression effects from the most abundant peak, as result of ion cloud Coulombic interactions (space-charge).

## 1 Introduction

In a mass spectrum, the isotopic fine structure of a molecule is defined as the well-resolved individual peaks of this molecule representing every isotopologue.^1^ It covers all isotopic combinations of atoms in the molecule. The observation of isotopic fine structure can provide precise assignment of an unknown molecular formula from a complex mass spectrum.^2^ Main atoms of organic compounds include carbon, hydrogen, oxygen, nitrogen, sulfur, phosphorus, etc. Among them, isotopes such as ^12^C, ^1^H, ^16^O, and ^14^N are referred to as the main isotopes. They have approximately 99% or even higher abundance, and ^32^S has around 95% abundance.^3^ Except for phosphorus, each of these elements has additional, lower abundance heavy isotopes such as ^13^C, ^17^O, ^15^N, etc., each of which has a slight mass shift due to the differences in nuclear binding energies, which results in multiple isotopologue peaks for most of the isotopic peaks of most molecules. Isotopologues are molecules which share the same chemical formula and bonding arrangement of atoms but differ only in their isotopic composition, with at least one atom having a different number of neutrons than the parent. When the size of a molecule (increasing the number of atoms) is increased, it becomes more difficult to separate all isotopologues, as the isotopic peaks become more densely distributed due to geometrically increasing combinations of isotopic compositions. For closely spaced peaks, the isotopologues can coalesce into a single peak because of insufficient resolving power (RP). Generally, a *m*/*z* 1000 molecule requires a RP at full width half-maximum (fwhm, *m*/Δ*m*_50%)_ of 1–5 M depending on the elemental composition.^1^ In the isotopic fine structure of most small molecules (<^∼^1500 Da), the monoisotopic peak represents the molecule consisting of the main isotopes, which is the highest peak in the mass spectrum. Glutathione (C_10_H_17_N_3_O_6_S) is a small molecule with a monoisotopic neutral mass (^12^C_10_^1^H_17_^14^N_3_^16^O_6_^32^S) of 307.083806, the fine structure of which consists of a monoisotopic protonated peak (*m*/*z* 308.091083) and isotopic peaks with a large number of combinations of main isotopes and ^13^C, ^2^H, ^15^N, ^17^O, ^18^O, ^33^S, ^34^S, and ^36^S.

While it remains challenging for the majority of mass spectrometers to achieve ultrahigh RP, it is routine for Fourier transform ion cyclotron resonance mass spectrometry (FT-ICR MS) to achieve ultrahigh RP and mass accuracy. Modern FT-ICR MS systems at 7 telsa can offer an ultrahigh mass resolving power of 10,000,000.^4^ Orbitraps such as Fusion Lumos can also reach a high resolving power of 1 M at *m*/*z* 200 with less than 1 ppm mass accuracy.^5,6^ At higher magnetic field strength, it is more feasible to resolve the isotopic fine structure,^7^ and space charge and cell designs also influence the performance of these instruments to resolve and study such features.

**Figure 1:**
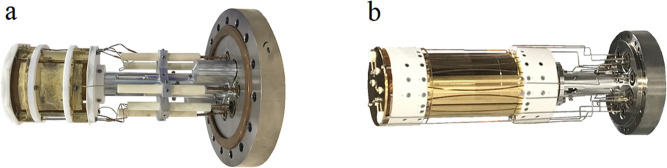
(a) Infinity cell; (b) dynamically harmonized cell (DHC).

The Infinity ICR cell (Figure 1a) concept is that a closed cylindrical cell with trapping plates at both ends can model the electric excitation field of an infinitely long cell.^8^ Linearizing the excitation field in this manner can greatly decrease ion loss along the *z*-axis compared to the corresponding open-cylindrical cell and, therefore, improve the sensitivity and enable longer transients and higher RP. But it also requires more delicate tuning at high performance. The dynamically harmonized ICR cell (DHC) (commercially marketed as the ParaCell) (Figure 1d) is a novel cell concept.^9^ It applies shaped electrodes and inherent trapping motion of the ions to achieve a parabolic trapping potential. The details and electric field of the DHC are described elsewhere.^10^ The ParaCell is able to stabilize the cyclotron motion of very low abundance ion clouds, allowing for the measurement of isotopic fine structure and the determination of the molecular formula for a wider dynamic range of ions.^2^ It can also stably excite ions to a larger orbit radius than the Infinity Cell, which in turn yields greater signal-to-noise and reduction in space-charge effects.^11^ Although not obvious at first glance, the DHC is also much simpler to tune for high performance. The details of the cell geometry designs can be found in a previous study.^12^

In this study, two FTICR MS instruments were applied to investigate the effects of different magnetic fields and the different ICR cell designs on RP and mass accuracy. In addition to the DHC, the 15 T FT-ICR MS is also equipped with 2ω detection. 2ω detection can detect at twice the usual frequency, as four cell plates are used for ion detection instead of the usual two.^13,14^ Hence, 2ω detection can significantly improve the instrument performance when other instrument conditions remain the same. Specifically, it can offer the equivalent RP in half of the detection time or double the RP in the same detection time.

## 2 Methods

### 2.1 Chemicals

A 5 μM glutathione solution (l-glutathione reduced, Sigma-Aldrich, Gillingham, UK) was prepared in 50:50 ultrapure water/methanol (VWR Co., Radnor, PA, USA) with 0.1% formic acid (Sigma-Aldrich Company Ltd., Dorset, UK). The standard tuning mix contains different compounds, such as hexamethoxyphosphazene or hexakis(2,2-difluoroethoxy)phosphazene, which gives a clean and evenly distributed mass spectrum of the mass range <3000 Da (Merck, Gillingham, UK). Ultrapure water was obtained from a Millipore Direct-Q purification system (18.2 Ω) (Merck Millipore, MA).

### 2.2 Instrumentation

The experiments were performed on two Bruker FTICR mass spectrometers (Bruker Daltonik, GmbH, Bremen, Germany) using a custom-built nanoelectrospray ionization (nESI) source. One is a 12 T (T) solariX FTICR MS with an infinity cell,^8^ and the other is a 15 T solariX 2XR FTICR MS with a dynamically harmonized ICR cell.^9^ Singly protonated glutathione ions were generated including the monoisotopic peak (*m*/*z* 308.091083), and other isotopic peak clusters were observed in broadband and narrowband (heterodyne mode) spectra and compared with simulated peaks by using the “simulpattern” tool in the DataAnalysis 5.0 software (Bruker Daltonik GmbH). Approximately 10–15 μL of the sample solution was loaded into a glass capillary tip, which was pulled by a P-97 Flaming/Brown micropipette puller (Sutter Instrument Co., Novato, CA), with a nichrome wire inside to provide the electrical connection.^15^ Nitrogen at 180 °C was used as the drying gas. A voltage of 600–800 V between the spraying tip and the capillary entrance was applied to facilitate the ESI process. The ions were isolated in the quadrupole mass selector with a *m*/*z* range of 20 and then accumulated in the collision cell for 0.08 s prior to transfer to the ICR cell (infinity cell/DHC) for excitation and detection. In both ICR cells (infinity cell & DHC), ions were either excited by a dipolar broadband excitation chirp (frequency sweep from 122 to 1000 Da) or excited and detected in a much narrower *m*/*z* range in heterodyne mode. The ion population in the cell was kept high enough to detect the fourth isotopic cluster (*m*/*z* 311) but low enough to minimize peak coalescence and ion–ion interactions. Details of the methods’ parameters can be found in Tables S1 and S2.

### 2.3 Data Analysis

All spectra were analyzed using DataAnalysis 5.0 software (Bruker Daltonik GmbH). Internal calibration was carried out to attain subppm assignment uncertainty. The peaks used for internal calibration are the monoisotopic peak and peaks with isotopes of ^15^N, ^13^C, ^34^S, ^18^O, and ^13^C^34^S. Data processing of 12 T FT-ICR MS used FTMSProcessing 2.2.0 software (Bruker Daltonik GmbH), and data processing of 15 T FT-ICR MS applied FTMSProcessing 2.3.1 software (Bruker Daltonik GmbH). In order to distinguish low-intensity peaks from the sidebands of adjacent high-intensity peaks, a full-sine window function was applied for apodization of the transient, which reduced the original RP by around 20–30%. It is reported that the RP can be doubled when the raw magnitude mode data is “phased” to produce absorption-mode data.^16−18^ However, in this work, only magnitude-mode data were used for discussion. A phase shift across the spectrum can prevent the absorption mode spectrum from being easily calculated. Hence, the absorption mode needs an accurate phase correction function,^17^ but producing an accurate phase function across a narrow *m*/*z* range is more difficult than producing the absorption mode spectrum across a full widely spaced spectrum with plenty of real ion (non-noise) peaks.^19^ In addition, as the 12 and 15 T instruments are sufficient to split the fine structure of glutathione, and the extra resolving power from using the absorption mode does not result in more information in this particular case.

## 3 Results and Discussion

### 3.1 Broadband Mode

**Figure 2:**
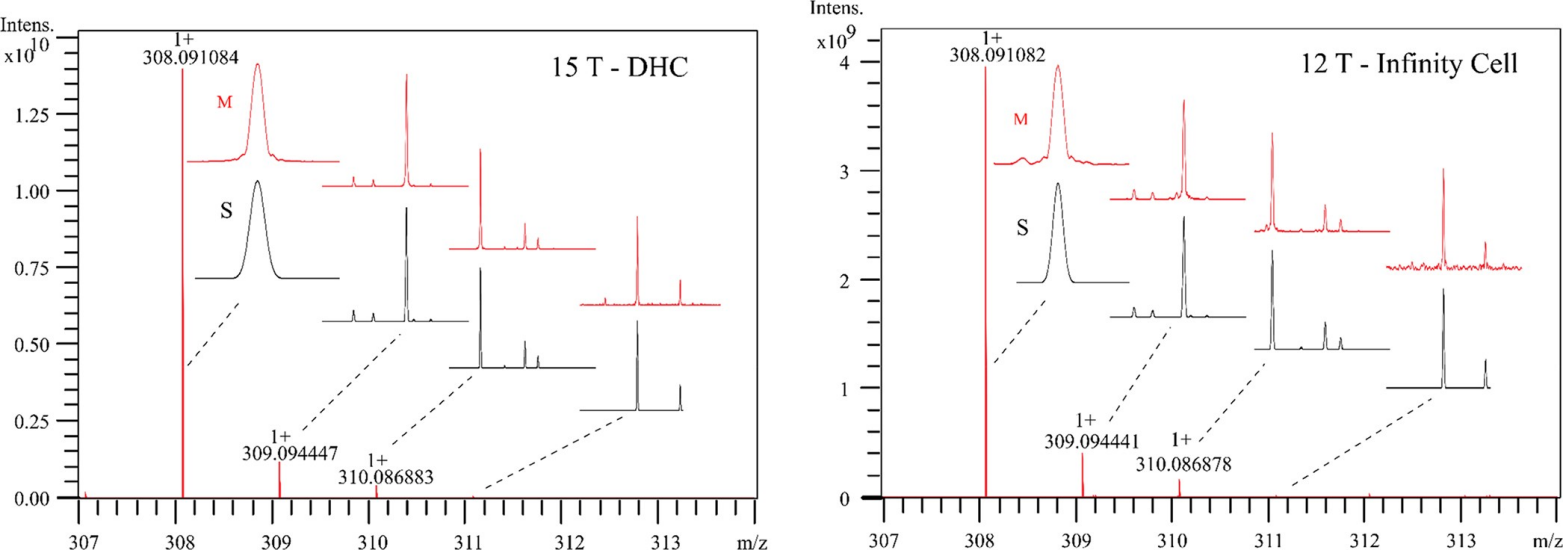
Complete pattern spectra of glutathione acquired in broadband mode using (left) 15 T with a Dynamically Harmonized ICR cell and (right) 12 T with an Infinity ICR cell. Black spectra marked with “S” are the simulated fine structure pattern, and the red spectra marked with “M” are the measured fine structure pattern.

**Figure 3:**
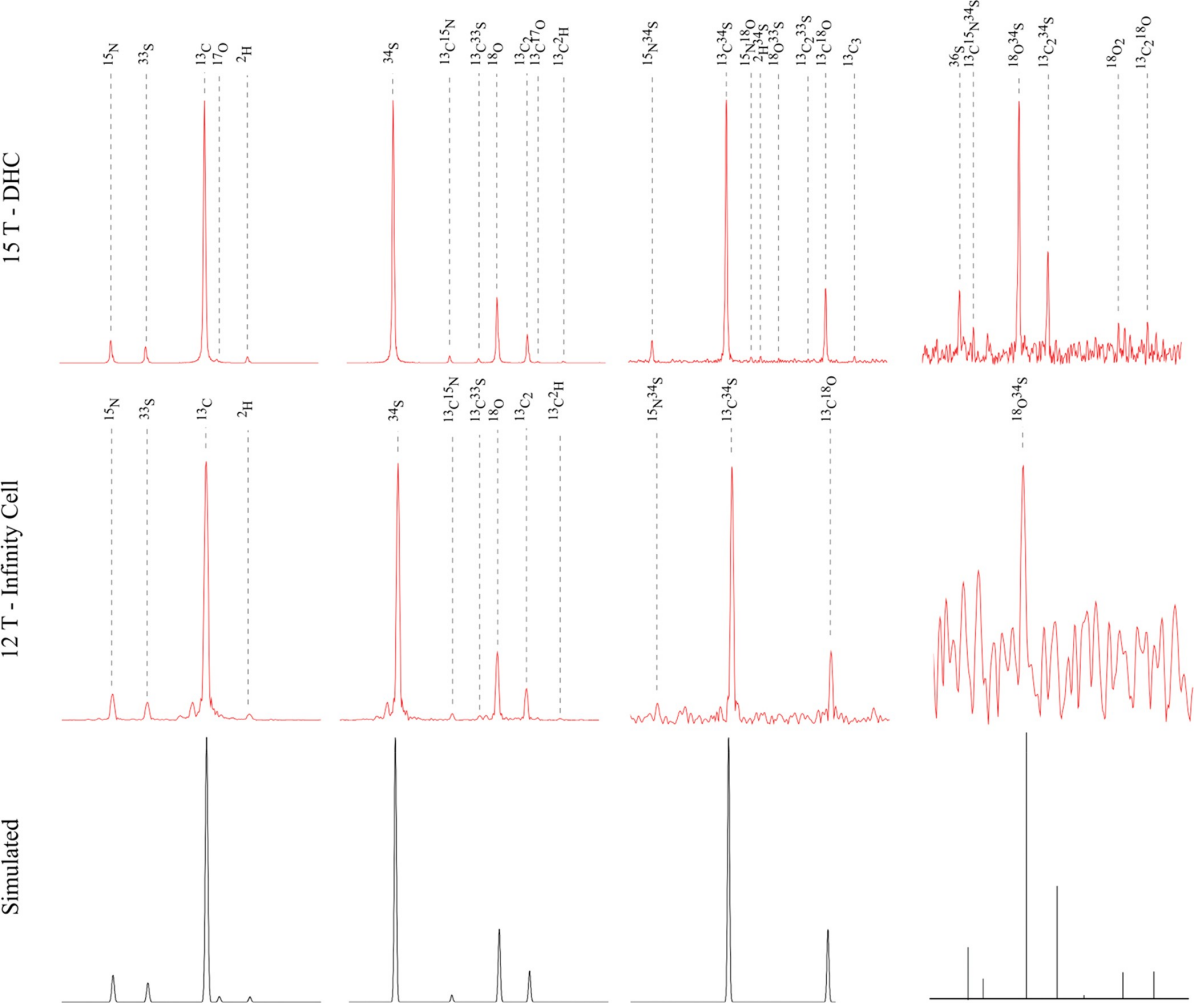
Magnification of the isotopic peak clusters of glutathione.

Figure 2 shows the complete fine-structure isotopic pattern spectra of glutathione containing the monoisotopic peak and the first three isotopic peak clusters of protonated glutathione at a RP of 1.96 and 1.00 M measured at the monoisotopic peak on 15 T with DHC (*m*/*z* range: 122.86–1000, data transient length: 4.4739 s, 16 M data points (32-bit)) and 12 T with infinity cell (*m*/*z* range: 122.84–1000, data transient length: 2.7962 s, 8 M data points (32-bit)), respectively. Figure 3 shows the magnification of the second, third, fourth, and fifth isotopic peak clusters of glutathione, with all peaks labeled with the heavy isotopes—as in Table 1. All *m*/*z* values and relative abundances of the clearly observed and resolved isotope peaks are listed in Table 1.

**Table 1:** *m*/*z* and Peak Abundance of the Measured and Simulated Isotopic Fine Structure Spectra of Glutathione with Resolving Powers of 1.96 and 1.00 M for the Monoisotopic Peak Measured by the 15 T with DHC^a^ and 12 T with Infinity Cell^b^, Respectively, Based on 100 Scans

				15 T-DHC					12 T-infinity cell				
	isotopic peak^c^	theor *m*/*z*	theor abundance (%)	measured *m*/*z*	measured abundance^d^(%)	mass accuracy (ppm)	S/N^e^	rel error of abundance^f^ (%)	measured *m*/*z*	measured abundance(%)	mass accuracy (ppm)	S/N	rel error of abundance (%)
1		308.091083	100.000	308.091084	100.000	0.0032	110869.2		308.091082	100.000	–0.0032	19519.7	
2	^15^N	309.088118	1.096	309.088116	0.691	–0.0065	764.4	32.0	309.088119	1.050	0.0032	203.0	3.0
3	^33^S	309.090471	0.790	309.09047	0.511	–0.0032	565.0	30.3	309.090472	0.716	0.0032	137.7	7.0
4	^13^C	309.094438	10.816	309.094447	8.665	0.0291	9604.7	15.6	309.094441	10.262	0.0097	2001.2	3.7
5	^17^O	309.095300	0.229	309.095273	0.116	–0.0874	127.0	46.1					
6	^2^H	309.097360	0.207	309.097353	0.214	–0.0226	234.7	2.2	309.097359	0.249	–0.0032	46.6	13.1
7	^34^S	310.086879	4.474	310.086883	3.097	0.0129	3431.4	25.7	310.086878	4.167	–0.0032	811.4	5.0
8	^13^C^15^N	310.091473	0.119	310.091472	0.084	–0.0032	90.9	24.6	310.09149	0.112	0.0548	19.8	4.3
9	^13^C^33^S	310.093825	0.085	310.093833	0.052	0.0258	55.8	33.9	310.093817	0.079	–0.0258	13.4	5.4
10	^18^O	310.095328	1.233	310.095328	0.800	0.0000	884.9	30.1	310.095323	1.109	–0.0161	214.6	7.5
11	^13^C_2_	310.097793	0.526	310.097791	0.346	–0.0064	381.2	29.3	310.097794	0.514	0.0032	98.3	1.7
12	^13^C^17^O	310.098655	0.025	310.098648	0.016	–0.0226	15.4	32.4					
13	^13^C^2^H	310.100714	0.022	310.100708	0.021	–0.0193	21.3	3.2	310.100643	0.034	–0.2290	4.7	31.2
14	^15^N^34^S	311.083914	0.049	311.083918	0.029	0.0129	30.1	36.3	311.083889	0.038	–0.0804	5.3	18.8
15	^13^C^34^S	311.090234	0.484	311.090234	0.331	0.0000	364.5	26.6	311.090235	0.453	0.0032	86.4	4.7
16	^15^N^18^O	311.092363	0.014	311.092342	0.008	–0.0675	6.7	39.4					
17	^2^H^34^S	311.093155	0.009	311.093149	0.009	–0.0193	7.9	0.8					
18	^18^O^33^S	311.094716	0.010	311.09469	0.006	–0.0836	4.9	32.9					
19	^13^C_2_^33^S	311.097180	0.004	311.097189	0.005	0.0289	3.9	20.6					
20	^13^C^18^O	311.098683	0.000	311.09868	0.104	–0.0096	113.2	17.4	311.098661	0.126	–0.0707	22.7	3.6
21	^13^C_3_	311.101147	0.015	311.101131	0.009	–0.0514	7.7	37.1					
22	^36^S	312.086092	0.011	312.086053	0.011	–0.1250	10.1	0.2					
23	^13^C^15^N^34^S	312.087268	0.005	312.087231	0.006	–0.1186	4.2	8.1					
24	^18^O^34^S	312.091124	0.055	312.091107	0.038	–0.0545	40.0	25.9	312.091159	0.045	0.1121	6.8	13.7
25	^13^C_2_^34^S	312.093588	0.024	312.093569	0.016	–0.0609	16.1	26.9					
26	^18^O_2_	312.099573	0.006	312.099569	0.006	–0.0128	4.9	3.0					
27	^13^C_2_^18^O	312.102038	0.006	312.102031	0.006	–0.0224	5.0	4.3					
Average								22.5					8.7

a *m/z* range: 122.86–1000, data transient length: 4.4739 s, 16 M data points (32-bit).

b *m/z* range: 122.84–1000, data transient length: 2.7962 s, 8 M data points (32-bit).

c The isotope peak refers to each of the measured peaks in Figure 3 at +1 charge state. The corresponding isotopologue of the isotopic peak refers to the following formula ^12^C_10_^1^H_17_^14^N_3_^16^O_6_^32^S with the same number of main atoms being replaced with the isotopes, e.g., the isotopologue of the isotopic peak ^15^N is ^12^C_10_^1^H_17_^14^N_2_^15^N^16^O_6_^32^S.

d The measured abundance of the ion species is calculated in reference to the intensity of the largest peak in the fine structure spectrum (the monoisotopic peak) and then expressed in percentages.

e The signal-to-noise ratio S/N of each peak is calculated as S/N = *S*/5σ, where S is the peak height above the peak baseline, and the noise N is determined as 5σ, with statistically 99% of the noise values within this range (5σ).

f Relative error of abundance is calculated as the standard deviation of the theoretical and measured abundance, and then divided by their average and then expressed in percentages.

For a given *m*/*z*, its corresponding frequency is proportional to the strength of the magnetic field. For example, the corresponding Nyquist frequency of the *m*/*z* (122) under 15 T magnetic field is 1875 kHz, while that of the *m*/*z* (122) chosen under the 12 T magnetic field is 1500 kHz. As the Nyquist frequency dictates the length of transient for a given number of data points, when the data points are the same for both 15 and 12 T FT-ICR MS, the transient length is inversely proportional to the magnetic field. Based on the calculation of mass resolving power (*m*/Δ*m*_50%_) under low pressure,^20^ to achieve the same RP of a given *m*/*z*, the 15 T FT-ICR MS requires 25% less acquisition time compared to the 12 T. When the transient data size of the 15 T experiment is doubled compared to the 12 T in broadband mode, the RP measured at the monoisotopic peak is nearly double that of the 12 T FT-ICR MS.

Figures 2 and 3 show that the positions of the measured fine structure peaks (red) agree with the simulated isotopic distributions (black) for both cells. The A+1, A+2, and A+3 peaks were simulated by the Bruker software “Simulate Isotopic Pattern” with an isotope abundance threshold of 0.1% (default value). The A+4 peaks (line spectra) were simulated using “IsoSpec” with an isotope abundance threshold of 0.001%.^21,22^ The 15 T with DHC resolved and observed 12 more isotopic peaks than the 12 T with an infinity cell, which could be partially due to the DHC being better able to stabilize the cyclotron motion of very low abundant ion clouds,^2^ and the DHC can also excite ions to a larger stable orbit radius to yield greater signal-to-noise and reduced space-charge effects,^11^ as mentioned earlier. The 15 T with the DHC resolved the ^13^C, ^13^C_2_, and ^13^C_3_ peaks, but 12 T with the infinity cell did not resolve the ^13^C_3_ peak. Peak coalescence was found between the ^13^C and ^17^O peaks when the 12 T with infinity cell was used, which could be due to space charge effects or field inhomogeneities within the cell, etc. In Table 1, the mass accuracy/mass error was calculated as the deviation of the measured *m*/*z* value and theoretical *m*/*z* value of the ion species and then expressed in parts-per-million (ppm). The measured abundance of the ion species is calculated in reference to the intensity of the largest peak in the fine structure spectrum (the monoisotopic peak) and then expressed as a percentage. The relative error of the abundance was calculated as the deviation of the measured and theoretical abundance of the peak and expressed in percentages.

Table 1 shows that the mass errors of the measured and theoretical mass were ≤125 and ≤229 ppb for 15 and 12 T, respectively. The relative error of the measured and theoretical abundance of all peaks ranged between 0.2–46.1% (average: 22.5%) and 1.7–31.2% (average: 8.7%) for 15 and 12 T, respectively. An Orbitrap Exploris 480 mass spectrometer (MS) was applied to successfully resolve the isotopic fine structure at the A+2 peak in the peptide MRFA with isotope abundance accuracies that match the spectral fit. However, this was done with a single selected ion monitoring (SIM) scan with isolation width 50 Th and fixed ion time (IT) of 8 ms, and the mass accuracies of the peaks were mostly between 200 and 600 ppb level.^5^ Another Orbitrap Q Exactive Plus mass spectrometer was applied to monitor how different ion populations affect mass and spectral accuracy in different compounds at a resolving power of 140000 at *m*/*z* 200. It showed that the Orbitrap slightly underestimates relative abundances of ^13^C_2_ in caffeine around 12% and ^34^S_1_ in MRFA around 15%. The relative abundances measured in the Orbitrap ultimately depend on the number of ions injected into the mass analyzer.^23,24^ The effects of varying the resolving power were not investigated in the Muddiman study, and the mass accuracies for three compounds were 600–700 ppb.^23^ In a paper by Nikolaev et al., the fine structure of isotopic peak clusters in mass spectra of reserpine and substance P were measured using 7 T FT-ICR MS with a DHC.^3^ The mass accuracies of the fine structure peaks were ≤200 ppb, which are comparable with this study. Some deviations from the theoretical isotopic distribution were also observed in their study. The peak intensity differences between the measured and theoretical peaks were mostly in the range of 30–70%, which are higher than those peak abundance deviations in this study. In this work, the 15 T showed higher relative abundance error for many of the isotopologues than the 12 T, but the signal/noise value (S/N) of the most abundant peak is also 5.7× higher, suggesting space-charge is contributing to the deviations. However, it is important to note that the two cells are different and also have different preamplifier designs, so the S/N levels may not be directly comparable, but in many cases, the information on mass-to-charge ratio and relative abundance are enough for unambiguous assignment of the elemental formula.^3^

### 3.2 Narrowband/Heterodyne Mode

#### 3.2.1 Comparison of Narrowband Data

**Figure 4:**
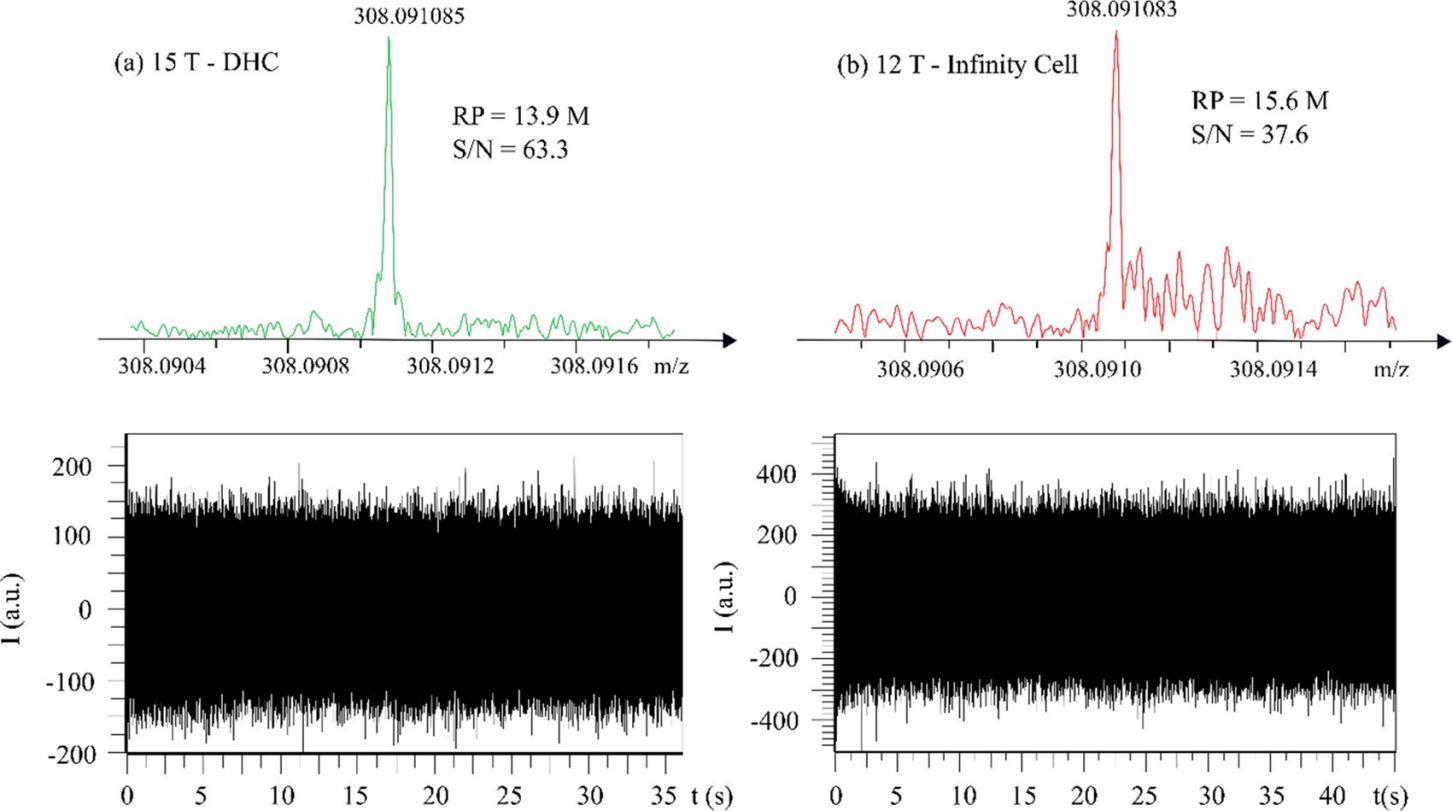
Highest resolving power observed for the monoisotopic peak of glutathione in narrowband (*m*/*z* 307–313) by (a) 15 T FT-ICR MS with a dynamically harmonized cell by 1-omega detection and (b) 12 T FT-ICR MS with an infinity cell. (Top) Mass spectra. (Down) Transient signal, single scan data shown.

Compared with broadband mode, which uses a wide frequency excitation, the advantage of narrowband detection is the increase of data points per frequency, which can result in much longer transient length and higher RP. Figure 4 presents the mass spectra and transient signal in narrowband mode with narrow mass range *m*/*z* 307–313 and data size of 1 M by both 15 T FT-ICR MS with a DHC and 12 T FT-ICR MS with an infinity cell. In this study, the recorded longest transient lengths of the 15 and 12 T were 36 and 45 s, respectively, with the corresponding highest RP observed for the monoisotopic peak of glutathione at 13.9 and 15.6 M, respectively. The 50 and 97 s transients were reported previously by a 9.4 T SolariX XR FT-ICR MS with a paracell in heterodyne mode.^25^ In this work, longer transient and higher RP can be achieved if the *m*/*z* window is set narrower. However, *m*/*z* 307–313 was applied here to include all possible isotopic peaks that can be detected by the instruments.

As the effective length and quality of the transient signals are limited by the space charge effects in the ICR cell,^26^ for the long transient, it is necessary to keep the ion populations relatively low in the cell to minimize these ion–ion charge-repulsion interaction effects. However, low ion populations will also result in low intensity of the detected ion peaks, which makes it difficult to observe less abundant peaks even with hundreds of accumulated scans. Therefore, for better comparison of fine structure data, the data size of 512 k in narrowband was used. The mass accuracies and measured abundance are summarized in Table 2.

**Table 2:** Narrowband Mass and Peak Abundance of the Measured and Simulated Isotopic Fine Structure Spectra of Glutathione with Resolving Powers of 7.5 and 7.6 M for the Monoisotopic Peak Measured by the 15 T with DHC^a^ and 12T with Infinity Cell,^b^ Respectively, Based on 10 Scans

				15 T-DHC (1ω)					12 T-infinity cell				
	isotopic peak	theor*m*/*z*	theor abundance (%)	measured *m*/*z*	abundance (%)	mass accuracy (ppm)	S/N	rel error of abundance (%)	measured *m*/*z*	abundance (%)	mass accuracy (ppm)	S/N	rel error of abundance (%)
1		308.091083	100.000	308.091083	100.000	0.0000	1048.4		308.09108	100.000	–0.0097	1768.5	
2	^15^N	309.088118	1.096	309.088117	1.013	–0.0032	8.6	5.6	309.088122	0.760	0.0129	11.5	25.6
3	^33^S	309.090471	0.790	309.090473	1.208	0.0065	10.6	29.6	309.090476	0.635	0.0162	9.3	15.4
4	^13^C	309.094438	10.816	309.094438	10.512	0.0000	108.1	2.0	309.094438	9.471	0.0000	165.9	9.4
5	^17^O	309.095300	0.229	309.095519	0.474	0.7085	2.9	49.3	309.095364	0.556	0.2071	7.8	58.9
6	^2^H	309.097360	0.207	309.097501	0.589	0.4562	4.1	67.9	309.097381	0.376	0.0679	4.7	41.0
7	^34^S	310.086879	4.474	310.086878	3.739	–0.0032	37.2	12.7	310.086877	3.371	–0.0064	57.7	19.9
8	^18^O	310.095328	1.233	310.095328	0.951	0.0000	8.0	18.3	310.095321	0.978	–0.0226	15.3	16.3
9	^13^C_2_	310.097793	0.526	310.097794	0.619	0.0032	4.5	11.5	310.097806	0.384	0.0419	4.8	22.1
10	^13^C^34^S	311.090234	0.484	311.090234	0.621	0.0000	4.5	17.5	311.090237	0.571	0.0096	8.1	11.7
11	^2^H^34^S	311.093155	0.009						311.092993	0.365	–0.5207	4.5	134.6
12	^18^O^33^S	311.094716	0.010						311.094866	0.347	0.4822	4.2	133.5
avg								23.8					44.4

a *m*/*z* range: 307–313, data transient length: 18.0355 s, 512 k data points (32-bit).

b *m*/*z* range: 307–313, data transient length: 22.5444 s, 512 k data points (32-bit).

**Figure 5:**
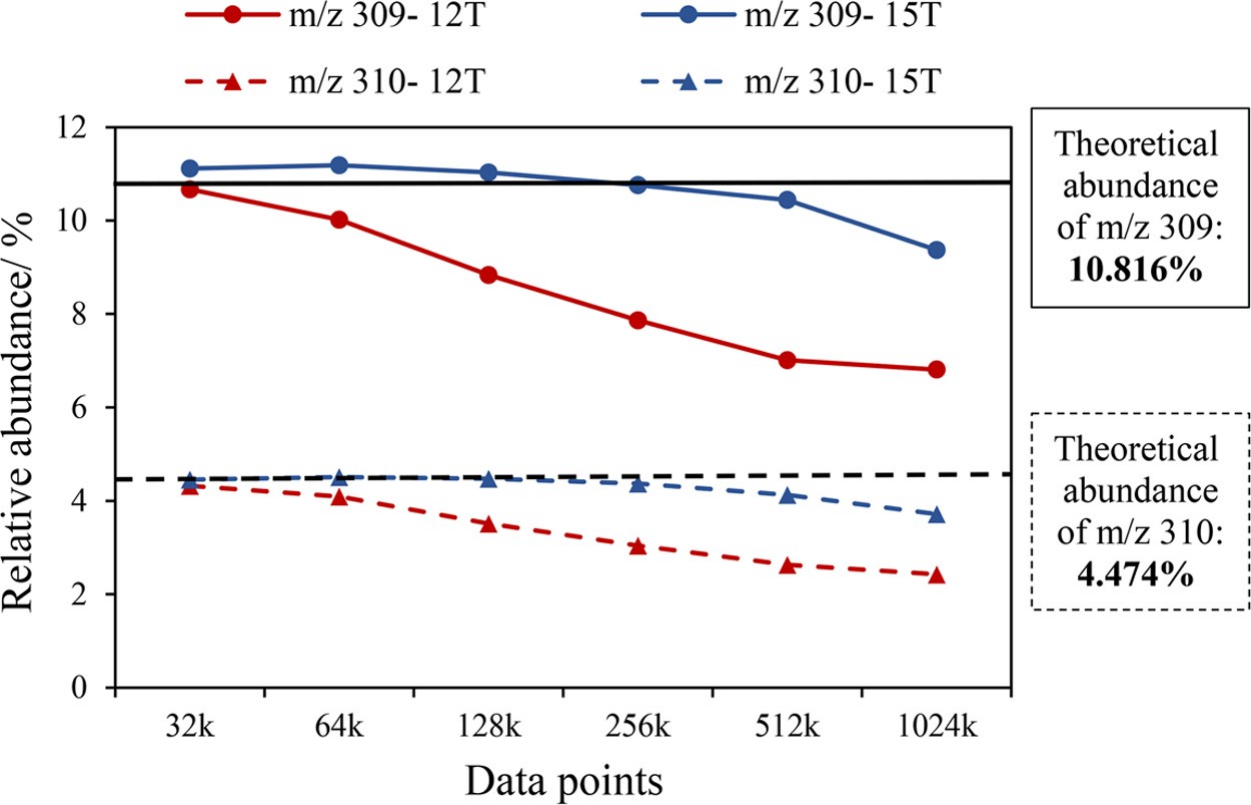
Relative abundance of the two most abundant isotopic peaks of glutathione measured by 12 T FT-ICR MS with an infinity ICR cell and 15 T FT-ICR MS with a dynamically harmonized ICR cell under different size of data points, based on 50 scans.

In Table 2, resolving powers of 7.5 M (15 T, transient duration: 18.0355 s) and 7.6 M (12 T, transient duration: 22.5444 s) are observed for the monoisotopic peak measured by the 15 T with DHC and 12T with infinity cell, respectively. The measured mass accuracies/errors were <709 and ≤521 ppb for the 15 T-DHC and 12 T-Infinity cell, respectively, though these mass accuracy values also include some very low intensity peaks which unsurprisingly also have the highest errors due to distortion of peakshapes by the noise, impeding peak analysis. The relative error of the measured and theoretical abundance of all peaks ranged between 2.0–67.9% (average: 23.8%) and 9.4–134.6% (average: 44.4%) on the 15 and 12 T, respectively, and the S/N values are comparable to minimize any differential space-charge effects between the two data sets. To investigate the possible effects of the resolving power on the relative abundance error, we compared the relative abundance of the two most abundant isotopic peaks of glutathione under different size of data points, as other peaks are hard to observe under lower resolving power. As shown in Figure 5, the relative abundance of *m*/*z* 309 and 310 are much lower than the theoretical abundance measured by the 12 T, and they approach the theoretical abundance values when the resolving power is decreased, as indicated by reduced data points. For the data obtained from the 15 T with the DHC, the relative abundance of *m*/*z* 309 and 310 are closer to the theoretical abundance. The relative abundance approaches the theoretical abundance when the data points are reduced from 1024 to 256 k, while they almost maintained at the theoretical values when the data points were reduced from 256 to 32 k. The data from both broadband and narrowband showed that a higher signal-to-noise ratio is preferable to assign more isotopic peaks. In narrowband, even though the RP is significantly improved, the low S/N leads to fewer assigned peaks than broadband. Therefore, more accumulated scans are needed to accurately assign more peaks. However, this will require delicate tuning of the cell and ions to minimize space-charge frequency shifts, especially during a very long transient. With very high resolution, on any Fourier Transform instrument, including the Orbitrap, signal averaging can be problematic if the peaks are shifting from scan-to-scan slightly due to space-charge frequency shifts.

As shown in Tables 1 and 2, among all measured isotopologues, those with theoretical abundance <1%, such as isotopologues with isotopes ^17^O, ^2^H, ^2^H^34^S, and ^18^O^33^S, are observed with much higher mass errors and relative abundance errors. This is possibly due to the small ion clouds in the ICR cell which can be strongly influenced by large ion clouds of similar *m*/*z* during the detection period, as these ion clouds are transiting through each other with a frequency equal to the difference of their cyclotron frequencies.^3,27,28^ This ion cloud interaction may have affected the relative positions and relative abundance of these peaks in the measured fine structure. In some extreme cases, this ion cloud interaction can cause peak coalescence,^29,30^ or totally/partially disperses the small ion cloud’s coherence, and lead to the decrease of peak intensity or completely eliminate the low abundance peak. To investigate this phenomenon, the three most abundant peaks are chosen and presented below.

#### 3.2.2 Peak Decay

**Figure 6:**
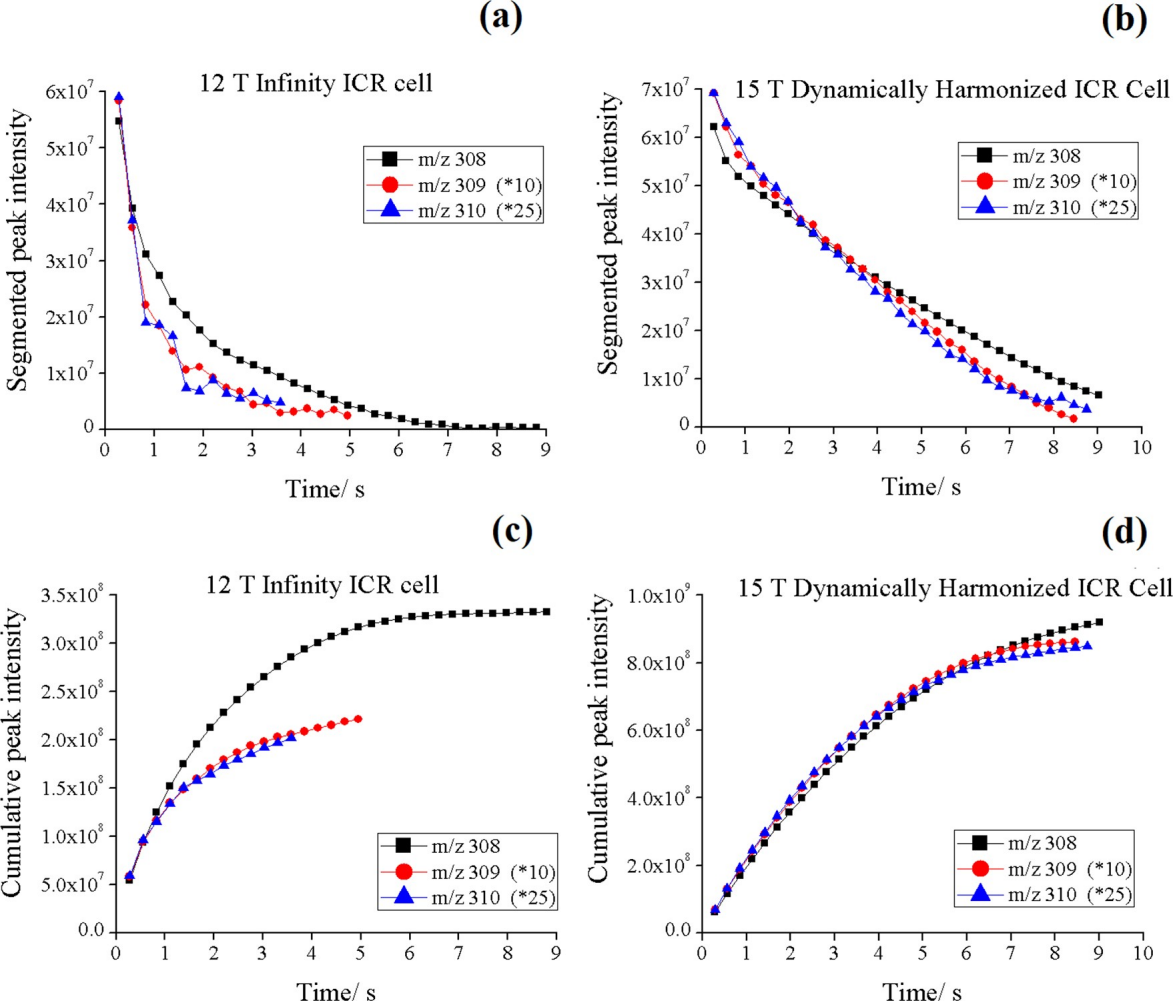
Segmented peak intensities of the three most abundant peaks of glutathione measured by (a) 12 T FT-ICR MS with an infinity ICR cell and (b) 15 T FT-ICR MS with a dynamically harmonized ICR cell; each point represent a segment of 1/32 of the total transient length. Cumulative peak intensities of the three most abundant peaks of glutathione measured by (c) 12 T FT-ICR MS with an infinity ICR cell and (d) 15 T FT-ICR MS with a dynamically harmonized ICR cell.

To compare this interaction phenomenon on both instruments, data with similar intensities and acquisition time were selected. The data was acquired in narrow mass range (*m*/*z* 307–313) with a data size of 256 k on the 15 T FT-ICR MS, while it was acquired in mass range of (*m*/*z* 294–324) with a data size of 1 M on the 12 T FT-ICR MS. The transient length was 9.0177 and 8.808 s for the 15 and 12 T, respectively, which was the most readily available compromise of parameters to provide very similar length transients and thus more comparable conditions. Each transient was divided into 32 equal segments. As it is difficult to observe the small peaks when the transient signal is divided into small segments, Figure 6 only plotted the segmented and accumulated peak intensities of the three most abundant peaks versus the acquisition time. To be more comparable in intensity with the *m*/*z* 308 peak, the peak intensities of the most abundant *m*/*z* 309 and 310 peaks are increased by 10- and 25-fold, respectively.

As shown in Figure 6a,b, the segment intensity of lower intensity peaks (*m*/*z* 309 and 310) decays faster than the higher intensity peak *m*/*z* 308 for both instruments. These peaks (*m*/*z* 309 and 310) also accumulated more slowly than the large peak *m*/*z* 308, as presented in Figure 6 c,d. This is most likely because the ion cloud interactions at the intensities of smaller peaks are generally suppressed by the more intense peak, and the minor ion “packets” are more vulnerable to the increase of any possible space charge interactions.^31^ The peaks measured by 12 T decay faster than those of the 15 T in the beginning, which may be due to space charge effects that resulted from insufficiently delicate tuning of the 12 T with an Infinity cell. Generally, to improve the sensitivity, ions are usually detected at ∼50% of the cell radius. However, electric field homogeneity only exists near the center of the infinity cell. Hence, the ions are either confined to small populations at the center of the cell which are subjected to space charge effects or the ions are excited to a larger orbit where the inhomogeneities can result in the loss of coherence.^32^ Thus, the infinity cell requires very delicate tuning to prevent coherence loss while maintaining sufficient sensitivity. In the DHC, the homogeneity of the electric field inside the cell allows the ions to be spaced out, which not only reduce the space charge effects but also increase the sensitivity, as the ions have larger orbit radius and closer to the detection plates. The DHC and/or the higher magnetic field may have also helped with preserving lower abundance ion cloud coherence. In addition, unlike the DHC, the center of gyration of the magnetron motion cannot be tuned/minimized via *x*–*y* deflection in the infinity cell as it can be in the DHC.

#### 3.3.3 2-Omega Detection

Apart from a normal dipolar 1-omega (1ω) detection on both 12 and 15 T FT-ICR MS, the DHC on the 15 T FT-ICR MS is also equipped with quadrupolar 2-omega (2ω) detection, which enables equivalent RP in half of the detection time or double the RP in the same detection time when other instrument conditions remain the same.^33^ The postcapture delay (PCD) curve was developed by Jertz et al. to study the behavior of the magnetron motion and quality of the centralization of both magnetron and cyclotron motion within the ParaCell by acquiring a series of FT-ICR spectra using varied PCD time intervals.^10^ The postcapture delay time refers to the time period that after the capture of the ions in the cell and before the start of cyclotron excitation of the ion. In this study, the delay time varied from 0 to 500 ms with 5 ms steps.

**Figure 7:**
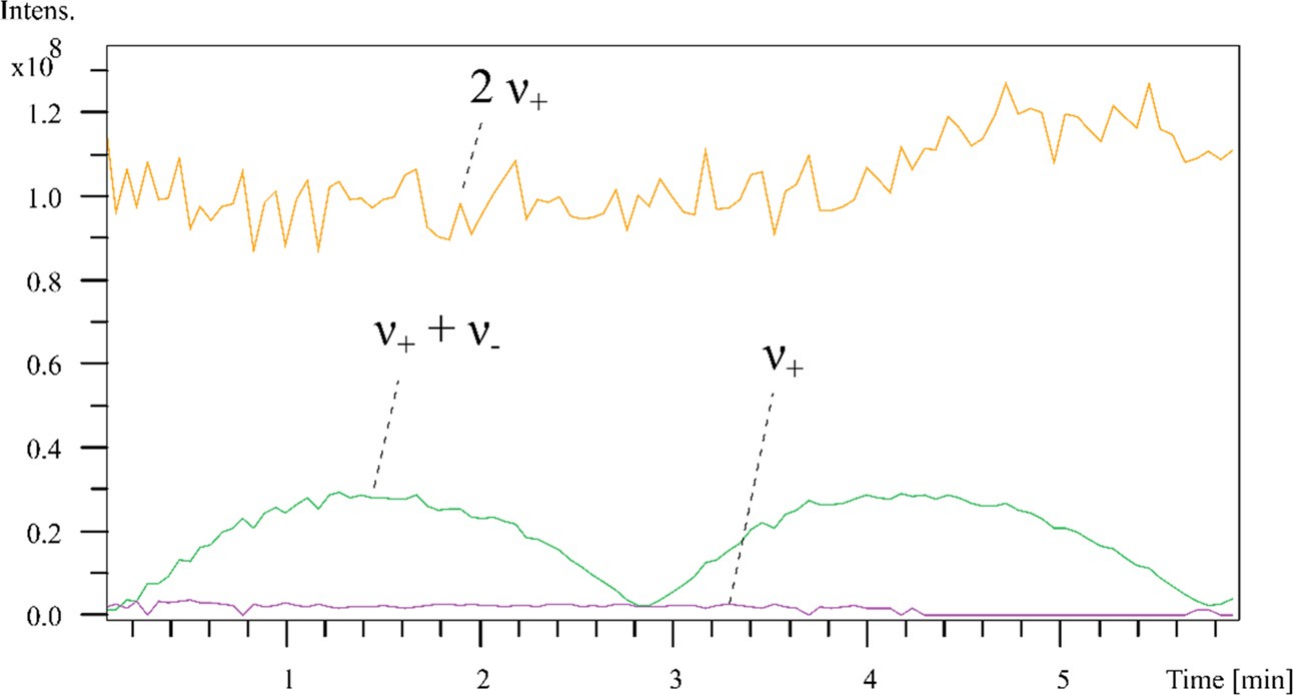
PCD curve measured by 15 T FT-ICR MS with dynamically harmonized cell in 2ω detection mode.

Figure 7 is an example of the postcapture delay (PCD) curve measured by 15 T FT-ICR MS with dynamically harmonized cell in 2ω detection mode. The peak with highest intensity (*m*/*z* 922.00968) of the standard tuning mix was chosen as the fundamental mass peak, and the relevant harmonic signals in quadrupolar 2ω detection appear at half of the frequency ν_+_ and ν_+_ + ν_–_, which is twice of the fundamental mass (ν_+_: 249, 828.56 Hz and *m*/*z* 1843.98379; ν_+_ + ν_–_: 249, 832.46 Hz and *m*/*z* 1843.95491) in 2ω mode.^34^ The difference between ν_+_ and ν_+_ + ν_–_ is the magnetron frequency ν_–_, which is 3.9 Hz. By plotting the relative intensity of ν_+_ and ν_+_ + ν_–_ versus the PCD time in each mass spectrum leads to an oscillating “PCD curve”. The position and height of minima and maxima of this PCD curve can be used to explain the size and the position of the magnetron orbit. In Figure 7, the 2 ν_+_ signal (orange line) represents the intensity variation of the fundamental mass peak under different PCD time. The two signals with frequencies ν_+_ and ν_+_ + ν_–_ represent the off-axis cyclotron motion and magnetron motion, respectively. As shown in Figure 7, the off-axis cyclotron motion (purple line) is very stable with intensities close to 0. The green line is a stable oscillating curve, representing the magnetron motion under different PCD time intervals. When the PCD time equals to 0, the intensities of the ν_+_ and ν_+_ + ν_–_ peaks are also close to 0, which suggest an acceptable tuning of the DHC.

**Figure 8:**
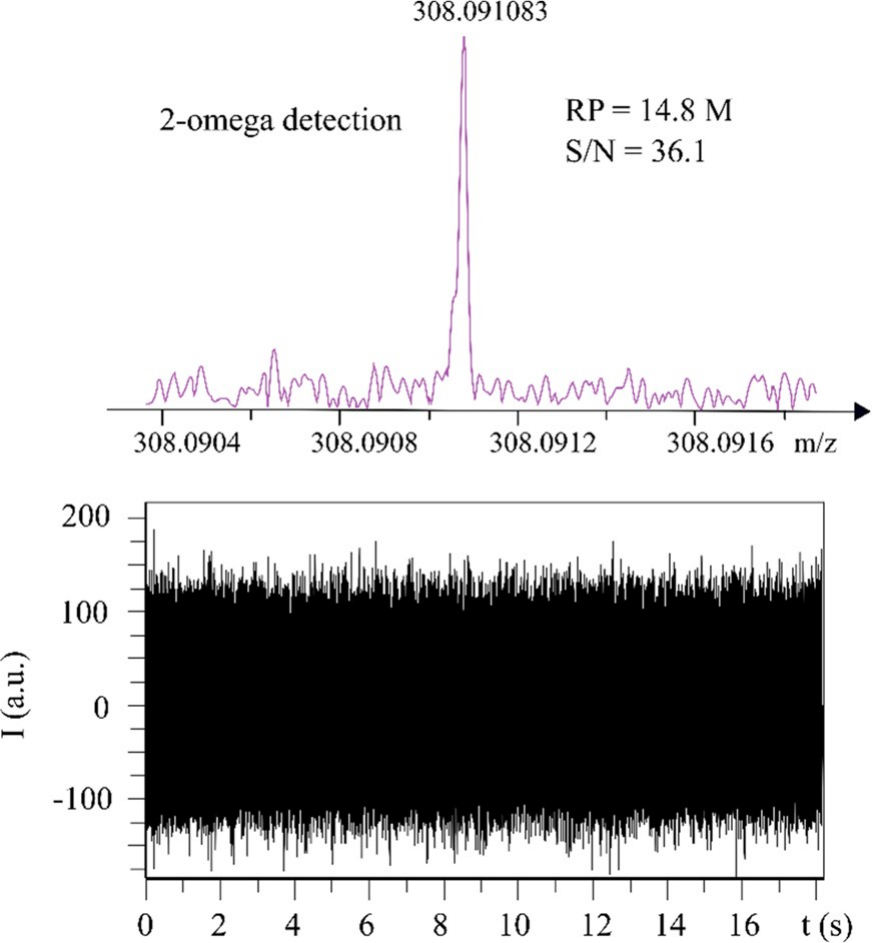
Highest resolving power observed for the monoisotopic peak of glutathione in narrowband (*m*/*z* 307–313) by 15 T FT-ICR MS with dynamically harmonized cell in 2ω detection mode. (Top) mass spectra; (down) transient signal, single scan data shown.

Figure 8 exhibited the highest RP observed so far for the monoisotopic peak of glutathione in narrowband (*m*/*z* 307–313) by 15 T FT-ICR MS with 2ω detection. It achieves an RP of 14.8 M within 18 s, which is only half of the acquisition time in 1ω detection (36 s).

## 4 Conclusions

The fine structure of isotopic peak distributions of glutathione in mass spectra was measured using Fourier transform ion cyclotron resonance mass spectrometry at 12 and 15 T magnetic field with an Infinity cell and a Dynamically Harmonized Cell, respectively. The positions of the measured fine structure peaks in the broadband agree with the simulated isotopic distributions with the mass error ≤125 and ≤229 ppb for the DHC and infinity cell, respectively. In heterodyne mode with a mass window of 6 *m*/*z*, we have shown that the highest RPs that can be achieved by FT-ICR MS are 13.9 and 15.6 M by the 15 T with DHC (transient length: 36 s) and 12 T with infinity cell (transient length: 45 s), respectively. Here, we demonstrated that the 2ω detection offers equivalent RP (14.8 M) in only half of the detection time (18 s). There is noticeable peak decay found for low abundance peaks, which may be the result of the suppression effects by the most abundant peak. The DHC was shown to be able to maintain detection of lower intensity species for a much longer time and with a more consistent decay constant between high and low intensity species than the infinity cell during comparable analysis.

Although this study provides a comparison of the DHC and infinity cell, it should be noted that this is not a direct comparison of two cells as there are other variables which also differ, such as different magnetic field, electronics, method parameters, etc.

## Supporting Information Available

Method parameters for the broadband and narrowband data (Tables S1 and S2) (PDF)
