# Supplementary material for: Fine Structure in Isotopic Peak Distributions Measured Using Fourier Transform Ion Cyclotron Resonance Mass Spectrometry: A Comparison between an Infinity ICR Cell and a Dynamically Harmonized ICR Cell
Source: J Am Soc Mass Spectrom. 2022 Jun 28;33(8):1499–509. doi: 10.1021/jasms.2c00093 (PMC9354249; doi:10.1021/jasms.2c00093)
Supplement: Supplementary file 1 — js2c00093_si_001.pdf [file js2c00093_si_001.pdf]

Supporting Information

Fine Structure in Isotopic Peak Distributions Measured using Fourier Transform Ion Cyclotron Resonance Mass Spectrometry: A comparison between an Infinity ICR cell and a Dynamically Harmonized ICR Cell

Jingsha Xu[1, 2], Meng Li[1], Bryan Marzullo[1], Christopher A. Wootton[1, 3], Mark P. Barrow[1], and Peter B. O'Connor[1*]

1. Department of Chemistry, University of Warwick, Coventry CV4 7AL, United Kingdom
2. Present address: Beihang Hangzhou Innovation Institute Yuhang, Xixi Octagon City, Yuhang District, Hangzhou 310023, China
3. Present address: Bruker Daltonics GmbH & Co. KG, Bremen, Germany.

*Corresponding author; Email: p.oconnor@warwick.ac.uk. Tel.: +44 (0)24 76151008

Table S1 Method parameters for the broadband and narrowband data presented in the study by the 12 tesla solariX FT-ICR MS (Bruker Daltonik, GmbH, Bremen, Germany) with an infinity cell

	**Broadband**	**Narrowband**		
		**1M**	**512K**	**Peak decay**
**Acquisition Mode**				
Acquisition Size (Megawords (MW)/ Kilowords (KW))	8MW	1MW	512KW	1MW
Low Mass Cutoff *(m/z)*	122.84	-	-	-
High Mass Cutoff *(m/z)*	1000	-	-	-
Narrowband Center *(m/z)*	-	310	310	309
Narrowband Mass Window *(m/z)*	-	6.01	6.01	29.38
Transient Length (s)	2.7962	45.0888	22.5444	8.808
Summed scans (n)	100	1	10	50
Ion Accumulation Time (s)	0.8	0.1	0.15	0.08
Ion Cooling Time (s)	0.25	0.5	0.5	0.25
**Source (Nano-ESI)**				
Capillary Voltage (V)	700	700	700	750
Capillary Exit (V)	200	200	200	200
Deflector Plate (V)	190	190	190	190
Funnel (V)	140	140	140	140
Skimmer (V)	12	12	12	12
Funnel RF (Vpp)	120	150	150	100

**Quadrupole**				
Q1 Mass (m/z)	310	310	310	308
Isolation Mass Window (m/z)	20	20	20	20
**Collision Cell**				
Collision Voltage (V)	0	0	0	0
DC Extract Bias (V)	0.1	0.1	0.1	0.1
RF Frequency (MHz)	2	2	2	2
Collision RF Amplitude (Vpp)	1400	1400	1400	1400
**Octupole**				
Frequency (MHz)	5	5	5	5
RF Amplitude (Vpp)	350	350	350	350
**Transfer Optics**				
Time of Flight (ms)	0.8	0.8	0.8	0.8
Frequency (MHz)	4	4	4	4
RF Amplitude (Vpp)	350	350	350	350
**Infinity Cell**				
Transfer Exit Lens (V)	-15	-15	-15	-15
Analyser Entrance (V)	-8	-8	-8	-8
Side Kick (V)	12.6	10.4	10.4	8.7
Side Kick Offset (V)	-6.9	-6.3	-6.3	-6.3
Front Trap Plate (V)	0.49	0.5	0.49	0.855
Back Trap Plate (V)	0.47	0.5	0.49	0.885
Sweep Excitation Power (%)	15	10	10	10

Table S2 Method parameters for the broadband and narrowband data presented in the study by the 15 tesla solariX XR FT-ICR MS (Bruker Daltonik, GmbH, Bremen, Germany) with a Dynamically Harmonized Cell (DHC)

	**Broadband**	**Narrowband**			
		**1M**	**512K**	**Peak decay**	**2ω**
**Acquisition Mode**					
Acquisition Size (Megawords (MW)/ Kilowords (KW))	16MW	1MW	512KW	256KW	1MW
Low Mass Cutoff *(m/z)*	122.86	-	-	-	-
High Mass Cutoff *(m/z)*	1000	-	-	-	-
Narrowband Center *(m/z)*	-	310	310	310	310
Narrowband Mass Window *(m/z)*	-	6	6	6	5.96
Transient Length (s)	4.4739	36.0710	18.0355	9.0178	18.1753
Summed scans (n)	100	1	10	100	1
Ion Accumulation Time (s)	0.08	0.02	0.06	0.04	0.08
Ion Cooling Time (s)	1.0 E^{-7}	0.25	0.25	1.0 E^{-7}	0.25
**Source (ESI/Nano-ESI)**					
Capillary Voltage (V)	4500	600	550	550	575
End Plate Offset (V)	-500	-	-	-	-

Capillary Exit (V)	200	200	200	200	200
Deflector Plate (V)	220	220	220	220	220
Funnel (V)	150	150	150	150	150
Skimmer (V)	15	15	15	15	15
Funnel RF (Vpp)	100	150	150	150	150
**Quadrupole**					
Q1 Mass (m/z)	120	310	310	310	310
Isolation Mass Window (m/z)	-	20	20	20	20
**Collision Cell**					
Collision Voltage (V)	0	0.0	0.0	0.0	0.0
DC Extract Bias (V)	0.6	0.4	0.4	0.6	0.8
RF Frequency (MHz)	2	2	2	2	2
Collision RF Amplitude (Vpp)	1200	1200	1200	1200	1200
**Octupole**					
Frequency (MHz)	5	5	5	5	5
RF Amplitude (Vpp)	350	350	350	350	350
**Transfer Optics**					
Time of Flight (ms)	0.8	0.8	0.8	0.8	0.8
Frequency (MHz)	4	2	2	2	4
RF Amplitude (Vpp)	350	350	350	350	350
**Para Cell**					
Transfer Exit Lens (V)	-20	-20	-20	-20	-20
Analyser Entrance (V)	-10	-10	-10	-10	-10
Side Kick (V)	0.0	0.0	0.0	0.0	0.0
Side Kick Offset (V)	-1.5	-1.5	-1.5	-1.5	-1.5
Front Trap Plate (V)	3.0	4	4	3.5	3.35
Back Trap Plate (V)	3.0	4	4	3.5	3.35
Sweep Excitation Power (%)	20	15	15	12	11
**Shimming DC Bias**					
0° (V)	2.745	2.750	2.750	2.750	2.755
180° (V)	3.255	3.250	3.250	3.250	3.245
90° (V)	3.085	3.080	3.080	3.080	3.080
270° (V)	2.915	2.920	2.920	2.920	2.920
**Gated Injection DC Bias**					
0° (V)	1.000	1.500	1.500	1.500	2.200
180° (V)	5.000	4.500	4.500	4.500	3.800
90° (V)	2.700	1.500	1.500	1.500	4.000
270° (V)	3.300	4.500	4.500	4.500	2.000
